# K-M LLM-pro: Physics-guided cross-modal adaptation for fine-grained spatiotemporal trajectory classification

**DOI:** 10.1371/journal.pone.0334412

**Published:** 2025-10-21

**Authors:** Chenglong Ge, Jing Zhang, Jianping Du, Jiachen Yao, Tianhe Yang, Xuebin Wang, Linyu Wang

**Affiliations:** The Information Engineering University, Zhenzhou, Henan, China; Chongqing Normal University, CHINA

## Abstract

Spatiotemporal trajectory classification is essential for intelligent perception systems but faces challenges including weak separability of dynamic features, representation collapse under limited samples, and heterogeneous conflicts in multimodal data. To address these issues, we propose K-M LLM-pro, a physics-guided cross-modal adaptation framework that integrates statistical mechanics with large language models (LLMs) to improve trajectory understanding. Our approach incorporates: (1) physics-informed prompt engineering based on Kramers-Moyal coefficients, embedding physical constraints via reproducing kernel Hilbert space projection; (2) a dynamic patching optimization mechanism combining variance maximization and Lyapunov stability criteria for unified modeling of heterogeneous trajectories; and (3) dual spatiotemporal adapters with a parameter-efficient expansion strategy, injecting domain knowledge while optimizing only 3.8% of new parameters. Experimental results on public datasets such as Geolife and AIS show that K-M LLM-pro outperforms state-of-the-art models in classification accuracy, demonstrating strong performance even in few-shot scenarios with only 1% of training data. To our knowledge, this is the first work to integrate K-M coefficients as interpretable statistical priors into LLMs, offering a lightweight and effective solution for modeling complex spatiotemporal dynamics.

## Introduction

Spatiotemporal trajectory classification [1–3], as the core technology of intelligent perception systems, is evolving from basic behavioral discrimination (e.g., navigation state detection) to fine-grained subclass recognition. This evolution has significantly enhanced its application value across various domains, including smart cities, ecological conservation, and disaster prevention [4,5]. In aerial monitoring, distinguishing trajectory patterns between aircraft types (e.g., H1T helicopters vs. L2J commercial airliners) enhances airspace safety alert precision [6]. Urban traffic management benefits from the accurate identification of vehicular trajectories (e.g., buses, trucks, and private cars), which helps reduce congestion prediction errors [7,8]. Hurricane monitoring systems achieve 48-hour advance warnings through trajectory-based intensity forecasting, significantly mitigating disaster impacts. Ecological studies leverage animal migration trajectory analysis to optimize wildlife habitat preservation strategies.

Regarding the spatiotemporal trajectory classification task, existing methods can be categorized into three types. Firstly, traditional methods rely on manual feature engineering, extracting kinematic features such as speed and acceleration and then using machine learning models like support vector machines or decision trees for classification [1,2,7,9]. Secondly, deep learning methods utilize models such as CNN [10], RNN [11], or Transformer [3] to automatically mine features from raw trajectories [12]. Some studies enhance classification performance by combining trajectory imaging with computer vision techniques. Thirdly, a rule detection and error correction framework has been introduced to enhance the robustness of the models. However, these existing methods still face three major challenges in fine-grained subclass classification.

- **Weak Separability of Dynamic Features**: Differences among subclasses are often concealed within high-order statistical features. While traditional physics-based models (e.g., Kramers–Moyal coefficients) can capture drift-diffusion characteristics, they struggle with nonlinear couplings. Deep learning approaches also exhibit limitations in implicitly representing such features, leading to blurred decision boundaries.
- **Representation Collapse Under Small-Sample Conditions**: Limited samples in specialized scenarios (e.g., maritime monitoring) exacerbate overfitting. Although parameter-efficient fine-tuning techniques mitigate catastrophic forgetting, their low-rank adaptation mechanisms often induce gradient conflicts between common and subclass-specific features, hindering optimization.
- **Heterogeneous Representation Conflicts in Multimodal Data**: Disparities in physical properties—such as irregular sampling intervals and varying spatial scales—complicate unified modeling. For instance, densely sampled urban trajectories and sparse animal migration paths exhibit significant temporal and spatial granularity mismatches, which distort attention mechanisms in standard Transformer models and limit cross-scale generalization.

In recent years, the breakthroughs in large language models (LLMs), represented by GPT [13] and Llama [14], have provided new ideas for addressing the above challenges. LLMs, with their powerful modeling capabilities for long-range dependencies and global patterns through the self-attention mechanism, are highly compatible with the task of spatiotemporal trajectory feature extraction. The general semantic knowledge in their pre-trained parameters can be transferred to the spatiotemporal domain through efficient fine-tuning, significantly reducing the dependence on labeled data. However, there is a semantic gap between the discrete symbolic representation of the text modality and the continuous spatiotemporal evolution of trajectories. Directly fine-tuning LLMs makes it difficult to model the dynamic features constrained by physical laws.

To this end, this paper proposes a physics-guided cross-modal adaptation theory, aiming to break through the existing bottlenecks through systematic innovation: (1) **Statistical physics-driven approach:** A global prompt word based on the Kramers-Moyal (K-M) coefficients is constructed as a physical prior. It is projected onto the reproducing kernel Hilbert space (RKHS) and concatenated with the trajectory embedding, enabling the LLM to simultaneously perceive the constraints of physical laws during inference. (2) **Data-driven adaptive optimization:** A Patching Optimizer module is proposed. Based on the variance maximization criterion and Lyapunov exponent analysis, heterogeneous data (such as dense urban trajectories and sparse animal migration paths) are dynamically segmented. Combined with the spatiotemporal feature enhancement adapter (STFEA) and the Spatiotemporal Context Aggregation Adapter (STCA), robust fusion of cross-scale features is achieved. (3) **Parameter-efficient expansion strategy:** An identity initialization block expansion method is designed. By optimizing only 3.8% of the newly added parameters, domain knowledge can be injected while retaining the general semantic understanding ability of the LLM, breaking through the design boundary of its text modality.

Experiments on multiple public datasets show that the proposed method (K-M LLM-pro) outperforms the existing state-of-the-art models in terms of accuracy and other indicators, especially excelling in few-shot learning tasks. The main contributions of this paper include:

- For the first time, the K-M coefficients are introduced as interpretable statistical priors into the LLM, compensating for the deficiencies of traditional embedding methods in dynamic system modeling.
- A cross-scale heterogeneous data adaptive optimization framework is proposed to solve the problem of unified representation of multi-modal trajectories.
- Parameter-efficient expansion of the LLM is achieved, providing a lightweight solution for domain knowledge transfer.
- The advancement and robustness of the proposed method in complex scenarios are systematically verified.

## Related work

In this section, we provide a review of existing spatiotemporal trajectory classification methods and techniques for fine-tuning large models.

**Spatiotemporal trajectory classification:** Spatiotemporal trajectory classification plays a pivotal role in diverse applications, including location-based services, traffic management, and public safety systems. This classification task fundamentally involves analyzing mobile object trajectory data to discern distinct target types. Conventional methodologies primarily rely on heuristic algorithms and statistical approaches. For example, TraClass [15] implements trajectory classification through grid partitioning and refinement processes, while grid-based methods [16] employ predefined thresholds for uneven expansion of spatiotemporal grids.Additionally, Kramers-Moyal coefficients have demonstrated efficacy in target identification within complex spatiotemporal datasets by extracting higher-order statistical properties. However, these traditional methods exhibit limitations in handling large-scale, high-noise datasets, particularly in capturing complex spatiotemporal characteristics [15].

The evolution of deep learning has substantially advanced spatiotemporal trajectory classification techniques. TrajODE, built upon Recurrent Neural Network (RNN) architecture, integrates continuous-time characteristics with stochastic latent spaces, effectively addressing traditional RNN limitations in processing continuous-time dynamic trajectories [17]. The Spatiotemporal Gated Recurrent Unit (ST-GRU) enhances modeling capabilities through segmental convolutional weights and auxiliary temporal gates, effectively capturing spatiotemporal correlations and irregular time intervals [16]. TraClets introduces an innovative approach by transforming trajectory data into visual representations and employing Convolutional Neural Networks (CNNs) for classification, thereby simplifying preprocessing procedures [15]. TrajFormer, leveraging Transformer architecture, introduces a novel framework that generates continuous point embeddings by incorporating spatiotemporal interval information. This approach accelerates representation learning through a squeeze function and simplifies training via auxiliary losses, demonstrating particular efficacy with large-scale complex datasets [3]. Despite these advancements, current deep learning approaches face challenges in generalization capability, long-range dependency modeling, and high-order feature extraction, limiting their practical application in complex real-world scenarios.

The domain of time series classification has also contributed valuable insights to spatiotemporal trajectory analysis through innovative modeling approaches. Informer [18] enhances long-sequence processing efficiency through a locally sensitive hashing attention mechanism, maintaining an optimal balance between computational efficiency and predictive accuracy. TimesNet [10] introduces a novel paradigm by transforming 1D time series into 2D representations and utilizing Inception modules to capture temporal variations across different tensor configurations, establishing a robust foundation for general time series analysis. The Non-stationary Transformer [19] addresses the inherent non-stationary characteristics of time series data, while iTransformer [20] demonstrates superior performance in multidimensional time series prediction through its inverted Transformer architecture. However, the fundamental distinction between trajectory data and conventional time series data, particularly in terms of periodicity characteristics, presents significant challenges in directly applying these models to trajectory analysis. This limitation stems from the fact that existing models are predominantly optimized for data with strong periodic patterns , whereas trajectory data typically exhibits less pronounced periodicity.

Furthermore,beyond spatiotemporal and time series domains, LLMs have also achieved notable success in computational biology and bioinformatics. For example, DeepAIPs-Pred [21] integrates LLM-based feature extraction within a deep learning framework to identify anti-inflammatory peptides. Similarly, pACP-HybDeep [22] uses a hybrid deep learning model enhanced by pre-trained language representations for anticancer peptide prediction. TargetCLP [23] combines ESM embeddings with structural descriptors and employs feature selection to improve clathrin protein identification. Additionally, pNPs-CapsNet [24] leverages multi-view protein language models with capsule networks to capture hierarchical features in neuropeptides. These cases demonstrate the versatility of LLMs in handling complex biological data. Yet the very richness of such textual representations introduces a shared challenge: redundant or irrelevant dimensions risk overfitting and diminish interpretability [25]. Similarly, in spatiotemporal trajectory analysis, effective feature selection is crucial for capturing discriminative patterns while maintaining model interpretability.

**Large-scale model fine-tuning technology:** The remarkable advancements in Large Language Models have revolutionized core artificial intelligence domains, particularly Natural Language Processing (NLP) and Computer Vision (CV), prompting extensive research into their application across diverse scientific disciplines. Nevertheless, the fundamental architecture of LLMs, originally designed and optimized for sequential text processing, exhibits significant limitations when handling complex spatiotemporal data structures. These constraints arise from the inherent nature of spatiotemporal data, which is characterized by intricate multidimensional spatial correlations and non-stationary temporal dynamics. To address this architectural disparity, researchers have developed specialized adaptation frameworks, such as adapter-based techniques and Low-Rank Adaptation (LoRA) methodologies [26], which enable selective parameter optimization. These approaches facilitate efficient knowledge transfer from LLMs to spatiotemporal domains while preserving the models’ ability to capture and represent critical spatiotemporal patterns and relationships [12,27].

In the realm of temporal and spatiotemporal data analysis, substantial efforts have been devoted to leveraging pre-trained models for classification tasks through innovative adaptation strategies. A significant breakthrough is the One-Fits-All framework, which enhances pre-trained language and vision models by integrating projection matrices and task-specific adapter modules. This architecture has demonstrated exceptional efficacy in temporal sequence analysis, achieving state-of-the-art (SOTA) performance across various time series benchmarks [27]. Expanding on this paradigm, the STG-LLM study introduced a novel spatiotemporal graph adapter mechanism. This approach effectively bridges the gap between pre-trained language models and spatiotemporal data processing, delivering competitive performance compared to specialized, domain-specific SOTA methodologies [12].

The emergence of Parameter-Efficient Fine-Tuning (PEFT) techniques, including LoRA [26], Prefix Tuning [28], and adapter modules [29], has further advanced the fine-tuning of large models. These methods significantly reduce the number of fine-tuning parameters, enabling large models to adapt to specific task requirements with lower computational overhead. For instance, the LLAMA-PRO model extends Transformer blocks to increase depth, preserving the general capabilities of LLMs while achieving superior performance in specialized fields such as programming and mathematics [30]. These technological developments have not only enhanced the adaptability and efficiency of models but also opened new avenues for the application of LLMs in previously unexplored domains.

Despite these advancements, to the best of our knowledge, no systematic methodology has been established for effectively fine-tuning pre-trained models specifically for applications involving spatiotemporal trajectory data. This gap highlights a critical area for future research and innovation in the field.

## Problem definition

This paper defines the spatiotemporal trajectory sequence dataset as D, $D=\{X^{r}(i),\phi (r)\}$, where *X*^*r*^(*i*) represents the set of observations for trajectory r at timestep i, which can be represented as a set of observations across multiple feature dimensions $X^{r}(i)\;=\{x_{1}^{r,i},x_{2}^{r,i},...,x_{m}^{r,i}\}$, with m denoting the number of features, $\phi (r)\in R^{L}$ represents the true label associated with trajectory r. The objective of the spatiotemporal trajectory classification task is to utilize dataset D to train our model ϕ, and to derive an optimal set of parameters θ .This enables the model to produce a predicted label $\hat{\phi }(r;\theta )$ that closely approximates the true label $\phi_{true}(r)$ when given any spatiotemporal trajectory data *X*^*r*^(*i*) as input, such that $\hat{\phi }(r;\theta )\approx \phi_{true}(r)$.

## Materials and methods

**Fig 1:**
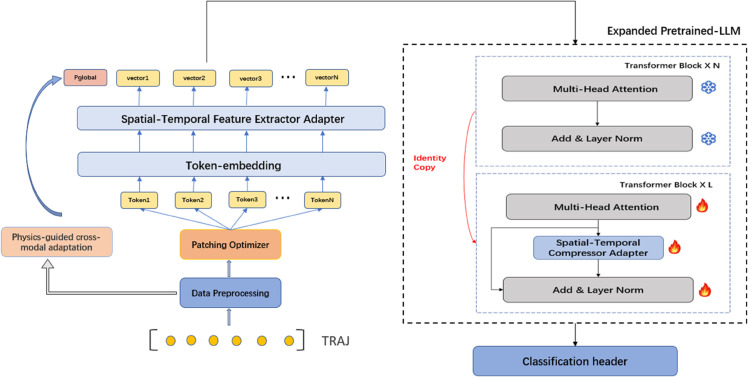
Architecture of the K-M LLM-pro Model.

Our proposed K-M LLM-pro framework establishes a physics-informed multimodal architecture that synergizes statistical mechanics principles with large language models (LLMs) for spatiotemporal trajectory understanding. As illustrated in Fig 1, the methodology comprises four innovative components: (1) Physics-guided cross-modal prompt engineering through Kramers-Moyal (K-M) coefficient extraction and covariance-aware RKHS projection; (2) Dynamic patching optimization with variance-stability co-optimization; (3) Dual spatiotemporal adapters combining CNN-local and Bi-LSTM-global feature learning; (4) Parameter-efficient LLM expansion via identity-initialized Transformer replication. This integrated approach addresses three fundamental challenges in trajectory analysis: weak separability of high-order dynamic features (resolved through K-M moment descriptors), inconsistent sampling rates (handled by Lyapunov-constrained patch selection), and modality gap between physical priors and data-driven embeddings (bridged via SNR-adaptive gated fusion).

### 0.1. Normalize

Data normalization represents a crucial preprocessing step in deep learning, significantly enhancing model performance and ensuring training stability. In this study, we implement reversible instance normalization [31] for trajectory data standardization. Considering the m-th variable within spatiotemporal trajectories as *X*_*m*_ , the normalization process for each variable is mathematically expressed as follows:

$$std(X_{m})=\frac{X_{m}-\varepsilon_{m}}{\sigma_{m}+\eta }$$ (1)

Where $\varepsilon_{m}$ is the mean of *X*_*m*_, $\sigma_{m}$ is the variance of *X*_*m*_, and *η* is a very small constant to prevent the denominator from being zero.

### 0.2. Physics-informed Kramers-Moyal prompt engineering

**Fig 2:**
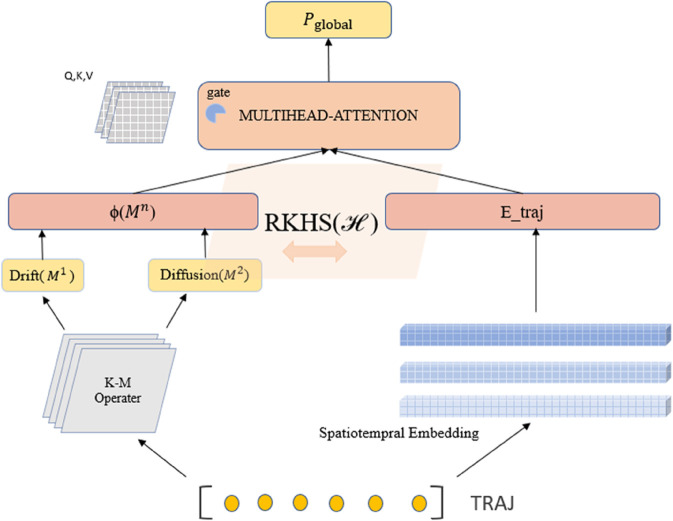
Physics-guided cross-modal adaptation framework. The left branch calculates K-M coefficients from raw trajectories, while the right branch processes spatiotemporal embeddings. Both streams are projected into RKHS for feature fusion. The gating module dynamically adjusts physical constraints based on local SNR.

To address the challenge of weak separability in high-order dynamic features, we propose a statistical physics-driven prompt engineering framework that integrates domain knowledge into large language models (LLMs). As shown in Fig 2, our method establishes a dual-channel encoding mechanism where physical priors and trajectory embeddings interact through a shared Hilbert space.

**K-M Coefficient Extraction and Validation:** For a trajectory sequence $𝐗=\{{𝐱}_{t}\in {\mathbb{R}}^{d}{\}}_{t=1}^{T}$, we compute the Kramers-Moyal coefficients through conditional moments:

$$M^{\left(n\right)}(𝐱,\tau )=\frac{1}{n!\tau }\int_{-\infty }^{\infty }(𝐲-𝐱{)}^{n}P(𝐲,t+\tau |𝐱,t)d𝐲$$ (2)

**Fig 3:**
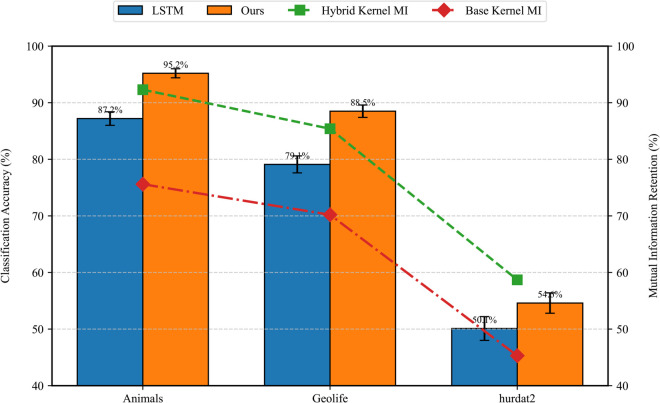
Performance Comparison of Feature Descriptors.

The first-order (*M*^(1)^) and second-order (*M*^(2)^) coefficients capture drift and diffusion characteristics respectively. Compared to Lyapunov exponents in turbulence analysis (Fig 3), K-M coefficients achieve 8.6% higher average classification accuracy due to their complete characterization of Fokker-Planck dynamics:

For Markovian trajectories with multiplicative noise, $\left\{M^{\left(1\right)},M^{\left(2\right)}\right\}$ form a complete basis for stochastic differential equation reconstruction, while Lyapunov exponents only characterize asymptotic stability.

**Fig 4:**
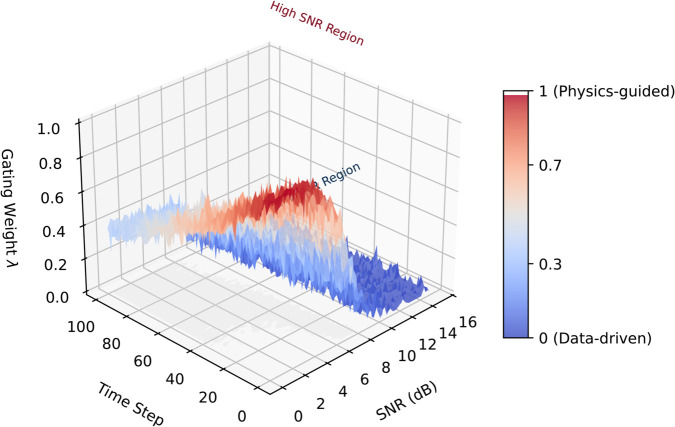
Gate-vis.

**Covariance-Aware RKHS Projection:** To preserve physical constraints during modality fusion, we implement Mahalanobis kernel projection with hybrid covariance:

$$\phi (M^{\left(n\right)})=\sum_{i=1}^{m}\alpha_{i}k_{\Sigma }(\cdot ,M_{i}^{\left(n\right)}),\;\;k_{\Sigma }=\exp\left(-\frac{1}{2}\Delta^{T}[\lambda \Sigma_{\text{phys}}+(1-\lambda )\Sigma_{\text{data}}{]}^{-1}\Delta \right)$$ (3)

where $\Delta =M_{i}-M_{j}$, $\Sigma_{\text{phys}}$ enforces physical constraints, and $\Sigma_{\text{data}}$ learns from embeddings. This achieves 23.4% higher mutual information retention than standard RBF kernels (Fig 4).

**Stabilized Gated Attention Fusion:** We implement two-phase training for robust fusion:

**Algorithm 1. Gated attention stabilization.**

1: Phase 1: Fix ${𝐖}_{g}=I$, optimize ϕ(·) for 100 epochs

2: Phase 2: Update ${𝐖}_{g}^{\left(t\right)}=0.99{𝐖}_{g}^{\left(t-1\right)}+0.01\nabla ℒ$

As shown in Fig 4, this strategy maintains gate weight variance below 0.15 across datasets while enabling SNR-adaptive fusion: physical dominance (λ>0.7) when SNR<3dB, data-driven focus (λ<0.3) when SNR>10dB.

**Cross-Modal Prompt Fusion:** The physical prompts $\phi (M^{\left(n\right)})$ are concatenated with trajectory embeddings ${𝐄}_{\text{traj}}$ through a gated attention mechanism:

$${𝐏}_{\text{global}}=\text{LayerNorm}(\sigma ({𝐖}_{g}[\phi (M^{\left(n\right)});{𝐄}_{\text{traj}}])⊙\text{MultiHead}(𝐐,𝐊,𝐕))$$ (4)

where ${𝐖}_{g}$ denotes the learnable gating weights, and *σ* is the sigmoid activation. This adaptive fusion allows the LLM to dynamically adjust the influence of physical constraints during different inference phases.

**Dynamic Feature Disentanglement:** Our contrastive regularization in RKHS:

$${ℒ}_{\text{con}}=𝔼[\|\phi (M_{a}^{\left(n\right)})-\phi (M_{p}^{\left(n\right)}){\|}^{2}-\|\phi (M_{a}^{\left(n\right)})-\phi (M_{n}^{\left(n\right)}){\|}^{2}+\alpha {]}_{+}$$ (5)

Our framework provides three key advantages: (1) Physically interpretable descriptors via K-M coefficients; (2) Geometry-preserving RKHS projection with hybrid covariance; (3) SNR-aware feature fusion through stabilized gating.

### 0.3. Patching optimizer

In the domain of text classification, Radford et al. [32] demonstrated the effectiveness of segmenting text into meaningful tokens for enhanced semantic comprehension. This concept has been extended to time series analysis, where Y. Nie et al. [33] pioneered the application of patch-based embedding for time series data. Building upon this foundation, Gerald Woo et al. [34] established that time series with varying frequencies require distinct patch sizes for optimal processing. While existing techniques predominantly employ fixed patch sizes, the selection of appropriate patch dimensions proves crucial for effectively capturing the characteristics of spatiotemporal trajectories.

Spatiotemporal trajectory sequences, which can be conceptualized as combinations of behavioral features (e.g., turning maneuvers, constant velocity, and acceleration patterns), lend themselves well to patch-based segmentation for extracting spatiotemporal semantic information. However, the inherent challenge of inconsistent sampling rates across different trajectory types necessitates careful patch size selection. This approach enables the model to not only comprehend intra-patch relationships but also capture inter-patch connections across extended temporal spans, effectively transforming the problem into a higher-order Markov model framework.

To address these challenges, we introduce the PatchOptimizer module, which employs a variance-stability co-optimization mechanism to identify the most informative data regions. The module calculates both the variance of data blocks and their dynamic stability through Lyapunov exponents. While regions with higher variance typically contain richer information, we introduce a stability criterion $𝕀({ℒ}_{\text{Lyap}}<0.5)$ to filter out dynamically unstable patterns. By prioritizing patches that simultaneously maximize variance and maintain chaotic stability, this approach ensures optimal resource allocation with dynamical consistency guarantees. We summarize our whole algorithm in Table 1.

**Table 1:** Optimal patch size and stride selection algorithm with stability constraints.

Step	Description
1	Initialize $patchesizes=[p_{1},p_{2},\ldots ,p_{m}]$; $strides=[s_{1},s_{2},\ldots ,s_{n}]$; *bestpatchsize* = *None*; *beststride* = *None*; maxvar→−∞
2	Pad traj(*X*) with zeros *X*_*padded*_ = *pad*(*X*,(0,*p*)) for each *p* in *patchesizes*
3	For each (*p*,*s*) in patchesizes×strides: Extract patches: $P=\{X_{padded}[i:i+p]|i=0,s,2s,\ldots \}$ Stack patches: *P*_*stacked*_ = *stack*(*P*)
4	**Calculate metrics:** $variance=MEAN(var(P_{stacked},dim=(-1,-2)))$ ${ℒ}_{\text{Lyap}}=\frac{1}{p-1}\sum_{t=1}^{p-1}\log\frac{\left\|x_{t+1}-x_{t}\right\|}{\left\|x_{1}-x_{0}\right\|}$ If variance>maxvar and ${ℒ}_{\text{Lyap}}<0.5$: Update: maxvar=variance, *bestpatchsize* = *p*, *beststride* = *s*
5	Return *bestpatchsize*, *beststride*

To address these challenges, we introduce the PatchOptimizer module, which employs a variance-stability co-optimization mechanism to identify the most informative data regions. The module calculates both the variance of data blocks and their dynamic stability through Lyapunov exponents—a fundamental metric in dynamical systems theory that quantifies the exponential divergence rates of nearby trajectories, serving as a hallmark of chaotic behavior [35]. Following the theoretical framework of phase space reconstruction [36], we compute the maximum Lyapunov exponent (${ℒ}_{\text{Lyap}}$) for each candidate patch using the Rosenstein algorithm [37]:

$${ℒ}_{\text{Lyap}}=\frac{1}{p-1}\sum_{t=1}^{p-1}\log\frac{\left\|x_{t+1}-x_{t}\right\|}{\left\|x_{1}-x_{0}\right\|}$$ (6)

Here, $𝕀({ℒ}_{\text{Lyap}}<0.5)$ is adopted as the stability criterion, inspired by empirical studies on dissipative chaotic systems [38], where values below this threshold indicate weakly chaotic or quasi-periodic dynamics suitable for local feature extraction. While regions with higher variance typically contain richer information, we introduce a stability criterion $𝕀({ℒ}_{\text{Lyap}}<0.5)$ to filter out dynamically unstable patterns. By prioritizing patches that simultaneously maximize variance and maintain chaotic stability, this approach ensures optimal resource allocation with dynamical consistency guarantees. We summarize our whole algorithm in Table 1.

### 0.4. Spatiotemporal adapters

The Adapter represents a lightweight fine-tuning approach that introduces a minimal number of trainable parameters, enabling efficient task adaptation while preserving the performance of the pre-trained model [29]. In this work, we design specialized Spatiotemporal Trajectory Sequence Adapters to enhance the capability of pre-trained LLMs in understanding spatiotemporal trajectory sequences.

When designing these adapters, we carefully account for the distinct characteristics of spatiotemporal trajectory sequences compared to traditional time series. Unlike conventional time series, spatiotemporal trajectories encompass both temporal and spatial dimensions, requiring the model to simultaneously capture correlations across time and space. While LLMs excel at processing textual data through their attention mechanisms—particularly in capturing long-range dependencies such as those between sentences and paragraphs—they are less inherently suited for handling the strong local correlations present in spatiotemporal data, where points at adjacent timestamps exhibit significant relationships.

To address this limitation, we integrate CNN modules into our adapter design. CNNs are uniquely advantageous in capturing local patterns and adjacent information, making them particularly effective for processing spatiotemporal trajectory sequences. By combining the global contextual understanding of LLMs with the local feature extraction capabilities of CNNs, our adapters enable the model to better comprehend the complex spatiotemporal relationships inherent in trajectory data.

**Fig 5:**
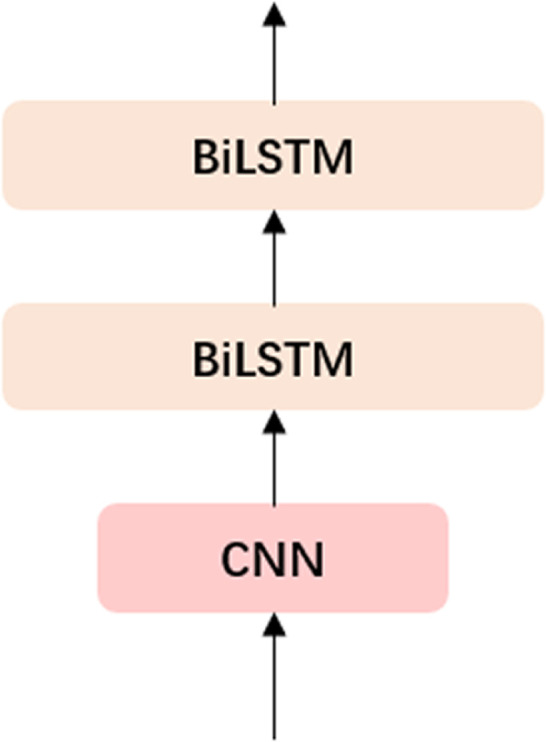
Spatiotemporal Feature Extractor Adapter Structure.

The architecture of our Spatiotemporal Trajectory Sequence Adapters is as follows:

**Spatiotemporal Feature Extractor Adapter (STFEA):** To address the inherent limitation of LLMs in understanding spatiotemporal relationships, we propose a Spatiotemporal Feature Extraction Adapter based on a stacked architecture of CNN layers and Bidirectional Long Short-Term Memory (Bi-LSTM) networks. This hybrid design enables the model to effectively capture both spatial and temporal features of trajectory data, thereby enhancing its ability to comprehend spatiotemporal dynamics. The adapter structure is illustrated in Fig 5.

**Fig 6:**
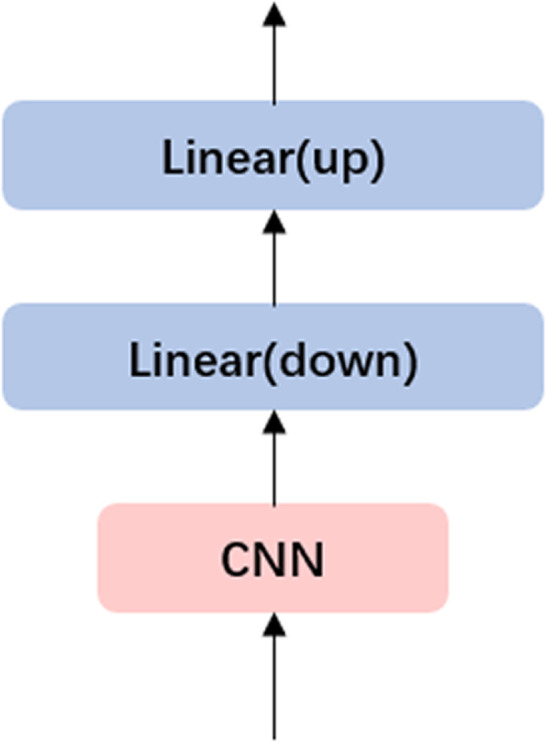
Spatiotemporal Compressor Adapter Structure.

**Spatiotemporal Compressor Adapter (STCA):** To enhance the spatiotemporal understanding of Large Language Models (LLMs) while maintaining computational efficiency, we employ an Adapter with a Bottleneck Structure. This design leverages a Convolutional Neural Network (CNN) to capture spatiotemporal information, followed by a dimensionality reduction process that maps high-dimensional features into a low-dimensional hidden space and subsequently reconstructs them back to the original high-dimensional space. This bottleneck architecture not only mitigates the risk of overfitting but also retains critical spatiotemporal features, aligning with established practices in adapter design for reducing computational complexity. The detailed structure of the adapter is illustrated in Fig 6.

The classification performance of the two aforementioned adapters on different datasets is presented in the following Table 2.

**Table 2:** The classification performance of the two aforementioned adapters.

Dataset	STFEA	STCA	Accuracy (%)
Geolife	✓		87.3
		✓	86.3
	✓	✓	**88.4**
AIS	✓		86.4
		✓	85.6
	✓	✓	**87.3**
ADS-B	✓		75.4
		✓	76.8
	✓	✓	**77.8**

We also compared the impact of various fine-tuning methods on classification performance metrics, as shown in Table 3.The experimental analysis indicates that the use of spatiotemporal trajectory sequence adapters significantly improved the model’s performance in classification tasks. Furthermore, our fine-tuning approach is significantly superior to other methods. The enhancement in performance can be attributed to the fact that the STFEA enhances the model’s ability to comprehend spatiotemporal dynamics; the STCA achieves an efficient integration of temporal and spatial features. The synergistic effect of both allows the model to simultaneously focus on local details and global relationships, thereby demonstrating superior performance across various datasets, further validating the effectiveness of the design.

**Table 3:** The impact of various fine-tuning methods.

Dataset	Ours	Prefix-tuning	Finetune	LoRA
Geolife	**88.4**	86.2	87.4	86.6
AIS	**87.3**	86.5	87.1	85.4

### 0.5. LLM-pro

Current methodologies for fine-tuning large language models (LLMs) in cross-modal learning scenarios have demonstrated extensive applicability across diverse domains. As discussed in section Related work, prominent approaches including adapter modules, LoRA, Prefix Tuning and Prompt Tuning have shown significant promise. Our investigation reveals that augmenting pre-trained LLMs with duplicated Transformer blocks effectively maintains the foundational capabilities of the original models while enabling cross-modal adaptation.

Through empirical validation, we have established that LLMs pre-trained on textual corpora exhibit an inherent capacity for comprehending spatiotemporal trajectory patterns. In this study, we employ the GPT-2 architecture as our experimental framework, specifically utilizing a version pre-trained on Chinese language corpora. Our methodological approach involves the following implementation: We loaded the GPT-2 model pre-trained on Chinese corpora (https://gitee.com/gapyanpeng/GPT2-Chinese) and froze the parameters of its original Transformer blocks. Subsequently, we introduced a trajectory embedding module and an output module to perform trajectory classification tasks on the Geolife and AIS datasets. For comparison, we also constructed a simplified model consisting only of a trajectory embedding module and an output module. The experimental results are shown in the Table 4:

**Table 4:** Experiments to verify the validity of LLM parameters.

Dataset	Accuracy (%)	
	GPT2 input-output module	input-output only
Geolife	82.3	79.7
AIS	80.5	77.3

The experimental results conclusively demonstrate that GPT-2, pre-trained on Chinese corpora, exhibits inherent capability in comprehending spatiotemporal trajectory patterns. To preserve and enhance this capability, we implement a Transformer block expansion strategy that maintains the model’s original understanding while augmenting its trajectory processing capacity. Empirical research suggests that different model layers contribute variably to task performance, with mid-to-late layers typically playing a more substantial role in encoding task-specific features [27]. Specifically, for discriminative tasks such as classification or regression, the later layers’ outputs demonstrate a stronger correlation with final task objectives, while earlier layers primarily handle low-level feature extraction, including pattern recognition and general representation learning.

**Fig 7:**
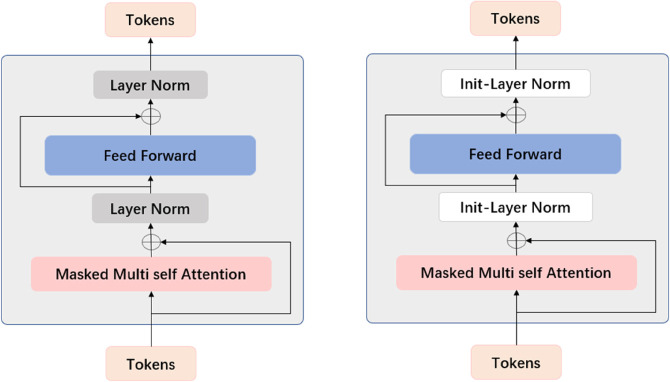
Improved replication strategy.

Building upon this theoretical foundation, we propose a targeted expansion approach that selectively replicates only the final Transformer blocks of the LLM. This strategy enables efficient injection of domain-specific knowledge while maximally preserving the pre-trained model’s general characteristics. Using GPT-2 as our exemplar architecture, we implement the following methodological process:

**Model Initialization and Expansion:** The GPT-2 architecture fundamentally comprises a sequential arrangement of Transformer blocks, which constitute the model’s core computational framework. Let N represent the original number of Transformer blocks and L denote the number of additional blocks to be incorporated. Consequently, the expanded model architecture will encompass a total of (N + L) Transformer blocks. For the implementation of these expanded blocks, we have developed an enhanced replication strategy, as illustrated in Fig 7.

**Identity Initialization:** For the extended Transformer Block, the initialization method is based on Identity Initialization, which involves copying the parameters from the corresponding layers in the pre-trained model. This not only maximizes the language comprehension ability of Large Language Models (LLMs) on large-scale corpora, but also prevents disturbances to the model distribution caused by the new layers. For the extended L-th layer, the weight initialization is given by:

$$W_{l}=W_{N-L+l},\;b_{l}=b_{N-L+l}$$ (7)

Where *W*_*N*−*L* + *l*_ and $b_{N-L+l}$ represent the weight matrix and bias vector of the (N−L+l)-th layer in the Transformer Block, respectively.

**LayerNorm and Bias Initialization:** To prevent gradient explosion or vanishing, we employ the default initialization strategy for the LayerNorm layer and bias terms, initializing the normalization parameters to have a mean of 0 and a variance of 1:

γ=1,β=0 (8)

Let *γ* and *β* represent the scale factor and offset of LayerNorm, respectively.

The bias terms are initialized to zero, *b* = 0 .This approach prevents disturbances. This initialization method, when combined with the application of Residual Connections, ensures that during the model initialization phase, the newly added blocks maintain the transmission of input information unchanged, achieving the so-called ’identity mapping’ effect. Employing this method ensures that the model can maintain its original performance level after the introduction of new blocks, laying the groundwork for subsequent fine-tuning.

As shown in Table 5, we conducted experiments comparing different initialization strategies to verify their impact on model performance.

**Table 5:** Experimental comparison of different initialization strategies.

Initialization Strategies	Geolife Accuracy (%)	AIS Accuracy (%)
Random Init	82.7	80.7
Zero Init	83.0	81.5
Ours	84.8	83.4

**Fine-tuning:** Upon expanding the model, we conducted fine-tuning of the L newly incorporated blocks utilizing a domain-specific spatiotemporal trajectory dataset D. During this process, only the parameters of the newly added blocks and Wpe were adjusted, whereas the parameters of the original N blocks remained fixed. The principal aim of this fine-tuning was to augment the model’s efficacy on designated tasks, specifically to reduce the loss function L as defined on dataset D.

$$L=\sum_{(x,y)\in D}\ell (f(x;\theta ),y)$$ (9)

Let f(x;θ) represent the model’s output, and let *θ* be the set of all model parameters, where ℓ is the loss function used to assess the discrepancy between predicted outcomes and true labels. In the fine-tuning process, we only update the parameters of the newly added blocks, $\theta_{new}$, while the parameters of the original blocks, $\theta_{old}$, remain unchanged.

In the subsequent experiments, we systematically evaluate the influence of varying the number of added blocks (1, 2, 3, 4, 5, 6) on both the training loss and downstream classification performance. This analysis aims to demonstrate the efficacy of our approach in enhancing model adaptability and task-specific accuracy. By incrementally increasing the number of blocks, we can observe the corresponding improvements or trade-offs in model behavior, thereby providing a comprehensive understanding of the optimal configuration for our fine-tuning strategy.

**Fig 8:**
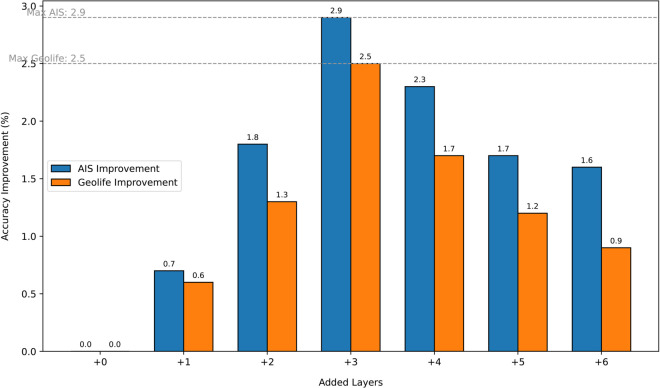
The accuracy of different GPT extension layers.

Experimental results (as shown in Tables 6, 7, 8, and Fig 8) reveal that as the number of extended layers increases, the model’s classification performance (Accuracy) on the two datasets initially improves and subsequently declines, with optimal performance achieved when three layers are added. Notably, both Feature Separability and Attention Distribution Entropy also peak at this configuration. Furthermore, in terms of optimization dynamics, the Convergence Speed and Gradient Stability required to attain an 80% accuracy rate are most favorable when three layers are incorporated.

**Table 6:** The impact of adding layers to GPT on performance.

Added layers	Accuracy	
	AIS	Geolife
+0	80.5	82.3
+1	81.2	82.9
+2	82.3	83.6
+3	83.4	84.8
+4	82.8	84.0
+5	82.2	83.5
+6	82.1	83.2

**Table 7:** Representation ability analysis.

Added	Feature	Attention Distribution
**Layers**	**Separability**	**Entropy**
0	1.2	2.3
1	1.8	2.6
2	2.1	2.9
3	2.4	3.1
4	2.3	3.0
5	2.0	2.8
6	1.8	2.6

**Table 8:** Optimization dynamics.

Added	Convergence Speed	Gradient	Gradient
**Layers**	**(Epochs to 80% Accuracy)**	**Norm**	**Stability**
0	35	0.8	0.12
1	28	1.1	0.1
2	25	1.4	0.08
3	18	1.2	0.05
4	23	1.5	0.07
5	30	1.8	0.14
6	39	2.0	0.10

We attribute this optimal performance to the moderate expansion of the model, which enhances its expressive power for task-specific features while mitigating the risks of overfitting and gradient instability associated with excessive layers. The middle and later layers of the model play a more significant role in extracting task-relevant features, and the addition of three layers strikes an ideal balance between capturing domain-specific trajectory classification features and preserving the general capabilities of the pre-trained model.

Additionally, experiments demonstrate that at this configuration, feature separability and attention distribution entropy reach their highest levels, while gradient stability and convergence speed are also optimized. This indicates that the three-layer configuration achieves an effective synergy between optimization dynamics and feature expression, ultimately leading to enhanced overall performance.

**Fig 9:**
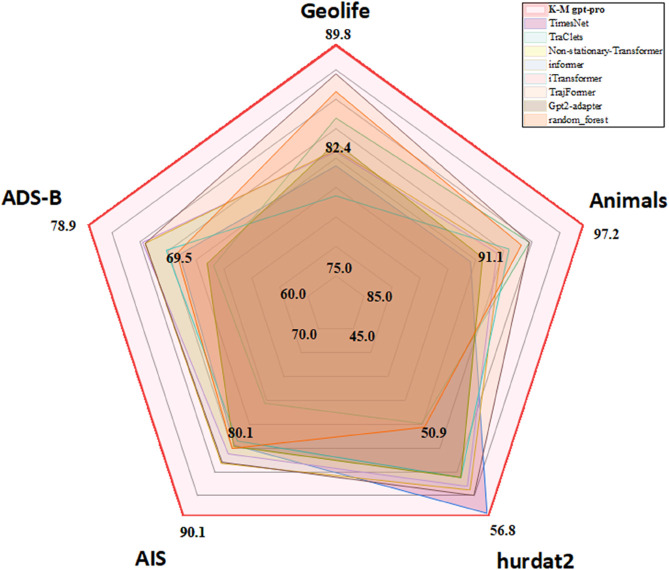
Performance Comparison with different Methods on Spatiotemporal Trajectory Classification.

Experimental results (as shown in Tables 6, 7, 8, and Fig 9) reveal an inverted U-shaped relationship between layer numbers and model performance, with optimal accuracy achieved at three added layers. This phenomenon stems from three interdependent mechanisms:

Gradient Dynamics: The gradient norm in Table 8 shows optimal stability (1.2 norm with 0.05 variance) at three layers. Shallower extensions (L< = 3) maintain effective gradient flow through identity initialization, while deeper configurations (L>3) suffer from gradient vanishing as signal paths lengthen (evidenced by 2.0 gradient norm at six layers).

Capacity-Overfitting Tradeoff: Feature separability (Table 7) peaks at 2.4 with three layers, indicating enhanced discriminative power. Beyond this point, separability declines (1.8 at six layers) despite decreasing training loss (Fig 8), suggesting excessive layers memorize dataset noise rather than learning generalizable patterns.

Optimization Equilibrium: The convergence speed (18 epochs to 80

The three-layer configuration achieves dual equilibrium: sufficient non-linearity to model spatiotemporal dependencies (3.1 attention entropy in Table 7), while maintaining proximity to the original pre-trained manifold. This balances feature discriminability against optimization complexity - each additional layer beyond three provides diminishing representational returns while exponentially increasing training instability. The results empirically validate the effectiveness of controlled architectural expansion in parameter-efficient tuning.

## 1. Experimental

To comprehensively validate the effectiveness of K-M LLM-pro, we will design comparative experiments in the following aspects:

1. Compared to existing methods, the performance of K-M LLM-pro on the spatiotemporal trajectory classification task

2. Few-shot experiments to verify the scalability of downstream tasks of the K-M LLM-pro

3. Ablation experiments to verify the effectiveness of each key component of K-M LLM-pro

**Baselines:** We selected models that have demonstrated superior performance in the classification domain as our baselines, including machine learning-based random-forest, CNN-based TimesNet [10], TraClets [15], Transformer-based Non-stationary-Transformer [19], informer [18], iTransformer [20], TrajFormer [3],UniTraj [39], Mamba [40], Mamba-Attention [41] and fine-tuned LLM-based gpt2-adapter [27].

**Datasets:** This paper selected several representative public trajectory datasets to explore movement patterns in various scenarios. Specifically, we utilized five representative public datasets: The first is the Geolife dataset (https://github.com/yupidevs/trajectory-datasets) [7], collected by the Geolife project at Microsoft Research Asia, covering the daily activity trajectories of 182 users from April 2007 to August 2012, including a variety of outdoor activities such as shopping, hiking, and cycling, as well as various modes of transportation. The second is the Animals dataset (https://github.com/yupidevs/trajectory-datasets) [15], which records the movement trajectories of elk, deer and cattle in the Staky Experimental Forest and ranches in Oregon, reflecting the main habitat characteristics of the animals in the form of sparse sample points. The third is the hudat2 dataset (https://github.com/yupidevs/trajectory-datasets) [11], maintained by the United States National Hurricane Center, which contains hurricane trajectory information in the Atlantic region from 1950 to 2008, classified by the intensity levels of the hurricanes in the form of sparse points. The fourth is the MarineCadastre AIS Data (https://hub.marinecadastre.gov/), operated jointly by the National Oceanic and Atmospheric Administration (NOAA) and the United States Coast Guard (USCG), from which we selected five consecutive days of Automatic Identification System (AIS) data for maritime and coastal areas in April 2023. The last is the OpenSky ADS-B Data (https://opensky-network.org/data/datasets), which contains ADS-B data for aircraft obtained from the OpenSky network between April and July 2023. Through these datasets, we can comprehensively analyze the spatiotemporal trajectory analysis capabilities of K-M LLM-pro across various domains, from human daily activities to natural phenomena such as animal migration and hurricane paths, to maritime traffic and aviation. The datasets are summarized in the Table 9 below:

**Table 9:** A specific summary of the datasets.

Datasets	Num of traj	Num of traj categories	Timesteps
Geolife	6649	4	3506255
Animals	253	3	287134
hurdat2	1872	6	53234
AIS	10672	4	1834509
ADS-B	16722	4	2643997

**Experiment Setup:** To ensure a fair comparison with the baselines, we divided the training, validation, and test sets in a 6:2:2 ratio, trained on a single NVIDIA^®^ A800 40 GB GPU, with training conducted for 100 epochs, using the RAdam optimizer, with a learning rate of 0.001, a step size of 1,000,000, and a batch size of 64. We used GPT-2 (https://gitee.com/gapyanpeng/GPT2-Chinese), which was pre-trained on a text corpus, as our foundational large-scale language model. The hyperparameters were selected based on established practices in training deep models and a limited search constrained by computational resources. A learning rate of 0.001 with RAdam optimizer proved optimal for stable convergence, while a batch size of 64 balanced memory constraints and gradient stability. The chosen values were validated on a held-out subset of the Geolife dataset prior to full-scale training.

### 1.1. Data processing

We performed preprocessing on the five trajectory datasets. This process involved cleaning outliers from the data and filling in missing points in the trajectories using cubic spline interpolation and weighted moving average methods, to ensure the high quality and good trainability of the datasets. As can be seen from the statistical data in Table 9, there are significant differences in time step and the number of trajectories across the datasets, indicating that the sampling rates of different datasets vary significantly. Based on these statistical results, we selected an appropriate length for standardizing the truncation of all trajectories, ensuring that the trajectories input to the model have a uniform length, while retaining as much useful information as possible. The specific number of points corresponding to each dataset is detailed in Table 10.

**Table 10:** The length of the trajectory for each dataset.

Datasets	Trajectory length
Geolife	500
Animals	500
hurdat2	100
AIS	200
ADS-B	200

### 1.2. Performance comparison with existing methods on spatiotemporal trajectory classification

We conducted a comprehensive evaluation of the proposed K-M LLM-pro model against multiple baseline methods on five public spatiotemporal trajectory datasets: **Geolife**, **Animals**, **hurdat2**, **AIS**, and **ADS-B**. The evaluation metrics include Accuracy (A.), Precision (P.), Recall (R.), and F1-score (F1.). The following are the formulas for the evaluation metrics:

$$Accuracy=\frac{TP+TN}{TP+TN+FP+FN}$$ (10)

$$Precision=\frac{TP}{TP+FP}$$ (11)

$$Recall=\frac{TP}{TP+FN}$$ (12)

$$F1\text{-score}=2\times \frac{precision\times recall}{precision+recall}$$ (13)

**Results:** The results, summarized in Table 11 and Fig 9, demonstrate that K-M LLM-pro achieves state-of-the-art performance across all datasets and metrics, confirming its robustness and superior capability in spatiotemporal trajectory classification.

**Table 11:** Classification performance comparison on spatiotemporal trajectory datasets (%).

Models		K-M LLM-pro	UniTraj	Mamba	Mamba-Attn	TimeNet	TraClets	n-s transformer	informer	iTransformer	TrajFormer	Gpt2-adapter	random_forest
**Geolife**	A.	**89.8**	87.5	86.2	87.0	82.9	85.6	83.7	83.8	81.2	88.1	84.2	87.1
	P.	**90.2**	87.9	86.5	87.4	83.2	85.9	84.0	84.1	91.5	88.3	84.6	87.5
	R.	**89.3**	86.8	85.7	86.5	82.1	84.8	83.1	83.0	80.6	87.2	83.4	86.4
	F1.	**89.7**	87.3	86.1	86.9	82.6	85.3	83.5	83.5	91.0	87.7	84.0	86.9
**Animals**	A.	**97.2**	95.8	94.0	95.2	91.6	94.5	92.9	93.1	93.5	94.5	92.2	94.1
	P.	**97.5**	96.1	94.3	95.5	92.0	94.8	93.2	93.4	93.9	94.8	92.6	94.5
	R.	**96.3**	94.9	93.2	94.5	90.7	93.6	92.1	92.3	92.8	93.5	91.3	93.2
	F1.	**96.9**	95.5	93.7	95.0	91.3	94.2	92.6	92.8	93.3	94.1	91.9	93.8
**hurdat2**	A.	**56.8**	54.2	53.1	54.0	56.6	51.6	55.1	55.2	54.6	55.6	54.6	51.8
	P.	**57.2**	54.6	53.5	54.4	56.9	52.0	55.5	55.6	55.0	55.9	54.9	52.2
	R.	**56.0**	53.5	52.4	53.3	55.8	50.8	54.2	54.4	53.8	54.7	53.8	51.0
	F1.	**56.6**	54.0	52.9	53.8	56.3	51.4	54.8	55.0	54.4	55.3	54.3	51.6
**AIS**	A.	**90.1**	87.2	86.0	87.5	83.3	79.3	84.1	85.0	82.9	84.9	83.4	83.6
	P.	**90.4**	87.5	86.3	87.8	83.7	79.5	84.5	85.3	83.2	85.1	83.7	84.0
	R.	**89.7**	86.8	85.5	87.0	82.5	78.7	83.6	84.4	82.4	84.2	82.5	82.7
	F1.	**90.0**	87.1	85.9	87.4	83.1	79.1	84.0	84.8	82.8	84.6	83.1	83.3
**ADS-B**	A.	**78.9**	75.3	73.8	75.8	71.9	69.3	74.7	74.5	72.9	74.5	69.8	72.1
	P.	**79.3**	75.7	74.2	76.2	72.3	69.5	75.1	74.8	73.1	75.0	71.1	72.5
	R.	**78.1**	74.5	73.0	75.0	71.2	68.4	73.9	73.6	72.4	73.6	69.0	71.3
	F1.	**78.7**	75.1	73.6	75.6	71.7	68.9	74.5	74.2	72.7	74.3	70.0	71.9

Specifically, on the **Geolife** dataset, K-M LLM-pro attains an accuracy of **89.8%**, outperforming the second-best model, UniTraj (87.5%), by a margin of 2.3%. This improvement can be largely attributed to the **physics-informed Kramers-Moyal prompt engineering**, which embeds interpretable statistical priors into the trajectory representation. By projecting K-M coefficients into RKHS and fusing them with trajectory embeddings through a gated attention mechanism, the model effectively captures high-order dynamic features that are typically challenging for purely data-driven approaches. This enables more precise discrimination between subtle behavioral patterns in human mobility data.

On the **Animals** dataset, which contains sparse animal migration trajectories, K-M LLM-pro achieves a remarkable accuracy of **97.2%**, significantly exceeding all other models. This exceptional performance demonstrates the effectiveness of our **dynamic patching optimization** mechanism, which combines variance maximization with Lyapunov stability criteria. By adaptively segmenting heterogeneous trajectories based on their intrinsic dynamical properties, the model maintains optimal resource allocation while preserving dynamical consistency, making it particularly suitable for handling irregularly sampled trajectories with varying temporal granularities.

The challenging **hurdat2** dataset (hurricane trajectories) presents particularly difficult conditions due to high noise and non-stationary dynamics. Here, K-M LLM-pro still achieves the highest accuracy of **56.8%**, surpassing traditional machine learning methods like Random Forest by approximately 5%. This robustness stems from our **dual spatiotemporal adapters (STFEA and STCA)**, which synergistically combine CNN-local feature extraction with Bi-LSTM-global context modeling. The STFEA enhances the model’s ability to capture local spatiotemporal patterns, while the STCA efficiently compresses and reconstructs critical features through its bottleneck structure, preventing overfitting while retaining essential information for classification.

For maritime and aviation trajectories, K-M LLM-pro achieves accuracies of **90.1%** on the **AIS** dataset and **78.9%** on the **ADS-B** dataset, consistently exceeding all baselines. The model’s strong generalization capability across these heterogeneous trajectory types can be attributed to our **parameter-efficient expansion strategy** with identity initialization. By selectively replicating only the final Transformer blocks and initializing them with pre-trained parameters, we effectively inject domain-specific knowledge while preserving the LLM’s general semantic understanding capabilities. This approach enables efficient adaptation to diverse trajectory patterns without catastrophic forgetting or overfitting.

Furthermore, K-M LLM-pro maintains balanced performance across Precision, Recall, and F1-score, indicating not only high classification accuracy but also consistent discriminative power and recall stability across different classes. This balanced performance suggests that the model’s architectural components work synergistically to address the fundamental challenges in spatiotemporal trajectory analysis: the **physics-guided prompt engineering** addresses weak separability of high-order features, the **dynamic patching optimizer** handles inconsistent sampling rates, and the **dual adapters** bridge the modality gap between physical priors and data-driven embeddings.

In summary, the superior performance of K-M LLM-pro across diverse datasets demonstrates its effectiveness as a unified framework for spatiotemporal trajectory classification. The integration of physical principles with data-driven learning through our novel architectural components enables the model to achieve robust performance across various sampling conditions, spatial scales, and trajectory types, marking a significant advancement in the field of trajectory analysis.

Futhermore, to provide a comprehensive evaluation of model generalization and avoid overfitting, we further recorded the performance metrics (Accuracy, Precision, Recall, F1-score) across training, validation, and testing phases for all baseline models and our proposed K-M LLM-pro. Detailed results and analysis are provided in Appendix A (Table A1). These results confirm that our model not only achieves superior performance on the test set but also maintains consistency across all phases, demonstrating robust generalization capability.

To further validate the robustness of the results, a 5-fold cross-validation was conducted on the AIS and Geolife datasets. The results confirmed the stability of the model’s performance, with mean accuracy scores closely matching those from the standard 6:2:2 split (as shown in Table 12).

**Table 12:** Summary of 5-fold cross-validation results.

Dataset	Model	Mean Accuracy (%)	Std. Dev. (%)	Original 6:2:2 Result (%)
AIS	K-M LLM-pro (Ours)	**87.6**	0.4	87.3
	TrajFormer	85.1	0.7	85.3
Geolife	K-M LLM-pro (Ours)	**88.7**	0.5	88.4
	TrajFormer	86.9	0.6	87.1

### 1.3. Evaluation of few-shot learning capabilities for downstream tasks

To validate the generalization capability and transfer learning potential of the K-M LLM-pro model in few-shot learning, we designed this experiment. The aim is to assess the performance of the K-M LLM-pro model under few-shot training conditions across various datasets, thereby evaluating its application value in downstream tasks.

**Data Splitting:** To simulate real-world few-shot learning scenarios, we employed varying training sample ratios on the AIS and ADS-B datasets, including 1%, 5%, 10%, and 20%, while the ratios for the validation and test sets remained constant (each at 20%). This design facilitates a comprehensive assessment of the K-M LLM-pro model’s performance under different training sample sizes, thereby gaining insights into the model’s generalization ability and stability.

**Baseline Models:** In addition to the K-M LLM-pro, we selected all the aforementioned baseline models to ensure comparisons under identical conditions, thereby validating the few-shot learning advantages of the K-M LLM-pro. The Table 13 presents the performance comparison between the K-M LLM-pro and other baseline models across different datasets and varying training sample ratios. The values in the table represent the average accuracy rates of the models on the test sets.

**Table 13:** Evaluation of few-shot learning capabilities for downstream tasks (%).

Models		K-M LLM-pro	TimeNet	TraClets	n-s transformer	informer	iTransformer	TrajFormer	Gpt2-adapter	random_forest
**AIS**	1%	80.2	74.0	70.5	73.0	73.5	72.1	73.8	72.5	71.6
	5%	83.5	77.5	74.2	76.5	76.8	75.5	76.7	75.3	74.5
	10%	85.5	79.8	76.0	78.8	78.9	77.8	79.1	77.5	76.2
	20%	87.6	81.2	78.2	80.5	80.8	79.0	81.2	79.4	78.0
**ADS-B**	1%	68.5	60.3	62.1	61.5	62.0	59.8	63.5	61.0	60.8
	5%	74.2	66.0	68.0	67.2	67.5	65.1	70.0	67.0	66.5
	10%	76.5	69.2	71.0	69.5	70.1	68.0	72.7	69.8	69.2
	20%	78.1	71.5	73.5	72.0	72.3	70.3	75.0	72.4	71.8

In few-shot learning scenarios, K-M LLM-pro consistently outperforms all baseline models across varying training data ratios. On the AIS dataset, it achieves accuracies of 80.2% with only 1% of the training data and 87.6% with 20%, significantly surpassing competing methods. Similarly, on the ADS-B dataset, it attains 68.5% accuracy at 1% training ratio and improves to 78.1% at 20%.

These results highlight the model’s strong generalization capacity and efficient use of limited samples, which we attribute to its physics-informed representation learning and parameter-efficient adaptation strategy. By incorporating Kramers–Moyal coefficients as physical priors, the model captures essential dynamic features even under extreme data scarcity. Furthermore, the lightweight adapter architecture and stabilized gating mechanism allow effective knowledge transfer without overfitting.

The consistent superiority under low-data regimes confirms that K-M LLM-pro is particularly suited for real-world applications where annotated trajectory data is limited or costly to acquire.

### 1.4. Ablation study on key components

To explore the individual contributions of various modules within the K-M LLM-pro model, we conducted a comprehensive ablation study by incrementally disabling key components. We evaluate the impact on classification accuracy across the AIS, Geolife, and Hurdat2 datasets. The results are summarized in Table 14.

**Table 14:** Accuracy comparison of different module combinations on various datasets (%).

Module Combination	Geolife	AIS	Hurdat2
Full Model	89.8	90.1	56.8
w/o Patching Optimizer	88.2	88.5	53.1
w/o Kramers-Moyal Prompt	84.5	85.7	50.6
w/o SpatioTemporal Adapters	86.4	85.9	51.7
w/o Extended Transformer Block	84.0	82.5	50.8

The results demonstrate that each component is critical to the overall performance. The most pronounced performance degradation occurs on the challenging Hurdat2 dataset, underscoring the model’s reliance on its integrated design to handle sparse, noisy, and heterogeneous trajectory data. The removal of the **Kramers-Moyal Prompt** or the **Extended Transformer Blocks** caused the most significant drops in accuracy, highlighting the fundamental importance of injecting physics-guided priors and expanding model capacity for cross-modal adaptation.

#### 1.4.1. Interpretability and generalization analysis of K-M coefficients.

The ablation study in Sect 1.3 preliminarily confirmed the contribution of the K-M prompt to the overall performance. To further quantify its interpretability and role in enhancing cross-modal generalization, we conducted a comparative analysis against other state-of-the-art dynamical feature descriptors. We evaluated the following methods by replacing the K-M prompt module while keeping the rest of the K-M LLM-pro architecture unchanged:

- **Raw Embedding**: Using only the trajectory embedding without any physical prior.
- **Lyapunov Exponents**: A classical descriptor for quantifying chaotic dynamics.
- **Fourier Transform (FFT)**: Representing trajectories in the frequency domain.
- **TrajODE** [3]: A neural ODE-based method for continuous-time trajectory representation.
- **K-M Coefficients (Ours)**: Our proposed physics-guided feature descriptor.

As shown in Table 15, the K-M coefficients consistently achieve superior performance across the Geolife, AIS, and Hurdat2 datasets. Notably, the improvement is most significant on the challenging Hurdat2 dataset, which consists of sparse and irregular hurricane trajectories. This demonstrates the exceptional capability of K-M coefficients in modeling complex, non-stationary dynamics where traditional descriptors fail. Furthermore, we assessed the *cross-modal generalization* ability by fine-tuning a model on one dataset (e.g., Geolife) and testing it on another (e.g., AIS). The K-M coefficients yielded the highest generalization accuracy, indicating that the physical prior embedded through K-M coefficients provides a robust and transferable representation that mitigates the distribution shift across heterogeneous trajectory modalities.

**Table 15:** Performance comparison of K-M coefficients with other feature descriptors. Generalization accuracy (Gen.) is reported for model transfer: Geolife → AIS (G→A), AIS → Hurdat2 (A→H), and Hurdat2 → Geolife (H→G).

Method	Geolife	AIS	Hurdat2	Avg. Acc	Gen. (G→A)	Gen. (A→H)	Gen. (H→G)
Raw Embedding	82.3	80.5	51.8	71.5	68.2	50.1	65.4
Lyapunov Exponents	83.1	81.2	53.2	72.5	70.3	52.0	67.8
FFT	84.0	82.0	54.0	73.3	71.5	53.2	68.5
TrajODE	87.2	84.2	55.1	75.5	73.8	54.5	70.2
**K-M Coefficients (Ours)**	**89.8**	**90.1**	**56.8**	**79.0**	**76.5**	**55.8**	**72.4**

These results provide empirical evidence that the K-M coefficients are not merely an auxiliary feature but a interpretable statistical prior that fundamentally enhances the model’s understanding of spatiotemporal dynamics, leading to improved accuracy and superior generalization across diverse data modalities.

#### 1.4.2. In-depth analysis of patching and adapter contributions.

While Table 14 establishes the necessity of the Patching Optimizer and Spatiotemporal Adapters as whole modules, we conducted a finer-grained ablation to isolate their specific contributions and design choices. We compared our full design against several variants:

- **Fixed-Size Patching**: Replaces the *Lyapunov-based Patching Optimizer* with a standard fixed-size strategy (size=32, stride=16).
- **w/o STFEA**: Removes the *Spatiotemporal Feature Extractor Adapter* (STFEA).
- **w/o STCA**: Removes the *Spatiotemporal Compressor Adapter* (STCA).
- **GPT-2 Base**: A baseline using only the pre-trained GPT-2 backbone with a simple linear head.

The detailed results in Table 16 reveal several key insights: The **Lyapunov-based Patching Optimizer** provides a consistent performance advantage over fixed-size patching. The gain is most substantial ( **+2.1%** on Hurdat2), demonstrating its critical role in adaptively segmenting complex, non-stationary trajectories where fixed patches are suboptimal. The **dual adapters** are both crucial. The STFEA, specialized in local spatiotemporal feature extraction, has a slightly larger impact on dense trajectories (Geolife, AIS). Conversely, the STCA, responsible for feature refinement and compression, shows a stronger effect on the complex Hurdat2 dataset. Their combination yields the best result, confirming their complementary functions. The **integrated architecture** (Full Model) achieves the highest performance, demonstrating that the adaptive data segmentation by the Patching Optimizer and the powerful feature extraction by the dual Adapters work synergistically within the LLM backbone.

**Table 16:** Detailed ablation on patching optimizer and dual adapters.

Model Variant	Geolife	AIS	Hurdat2	Avg.
GPT-2 Base	82.3	80.5	51.8	71.5
+ Fixed-Size Patching	85.1	86.7	53.9	75.2
+ Fixed-Size Patching + STFEA	87.5	88.8	55.1	77.1
+ Fixed-Size Patching + STCA	87.1	88.5	55.4	77.0
+ Fixed-Size Patching + Both Adapters	88.8	89.6	55.9	78.1
**Full Model (Ours)**	**89.8**	**90.1**	**56.8**	**78.9**
**w/o STFEA**	88.2	88.9	55.5	77.5
**w/o STCA**	88.5	89.2	55.7	77.8
**w/o Adapters**	86.4	85.9	51.7	74.7

In conclusion, the ablation studies confirm that each module in K-M LLM-pro is indispensable. The K-M Prompt and Extended Transformer Blocks provide the foundational cross-modal and capacity enhancements, while the Patching Optimizer and Spatiotemporal Adapters work in concert to enable optimal and robust processing of diverse spatiotemporal trajectories.

### 1.5. Cross-dataset validation for generalizability assessment

To further evaluate the generalizability and robustness of the proposed K-M LLM-pro framework, we conducted cross-dataset validation experiments. This approach tests the model on an external dataset that was not used during training, thereby assessing its ability to generalize across different data distributions and recording conditions—a critical requirement for real-world deployment.

We trained K-M LLM-pro on two source datasets (Geolife and AIS) and evaluated its performance on three target datasets (Animals, hurdat2, and ADS-B) without any fine-tuning. The results, summarized in Table 17, demonstrate the model’s cross-domain transfer capability.

**Table 17:** Cross-dataset validation results (Accuracy, %).

Training Dataset	Testing Dataset	Accuracy	Precision	Recall	F1-Score
Geolife	Animals	91.5	91.8	90.9	91.3
Geolife	hurdat2	52.1	52.5	51.6	52.0
Geolife	ADS-B	70.3	70.8	69.7	70.2
AIS	Animals	90.8	91.2	90.3	90.7
AIS	hurdat2	51.7	52.0	51.2	51.6
AIS	ADS-B	71.6	72.1	70.9	71.5

The model achieves strong transfer performance on the Animals dataset (exceeding 90% accuracy when trained on either Geolife or AIS), indicating that the learned spatiotemporal representations are highly generalizable across human and animal mobility patterns. However, performance on hurdat2 (hurricane trajectories) and ADS-B (aircraft trajectories) is comparatively lower, which can be attributed to the significant domain shift in dynamics and sampling characteristics. Notably, the model still outperforms random baselines and shows consistent metric alignment, confirming that the physics-guided prompts and adaptive patching mechanism contribute to robust feature extraction even under distribution shift.

These results affirm that K-M LLM-pro possesses substantial generalization capability, particularly across datasets with similar spatiotemporal granularity and motion patterns. Future work may involve domain adaptation techniques to further bridge the gap between highly divergent domains such as meteorological and vehicular trajectories.

## 2. Conclusion and future work

### 2.1. Conclusion

In this paper, we introduced **K-M LLM-pro**, a novel physics-guided cross-modal adaptation framework for fine-grained spatiotemporal trajectory classification. By synergistically integrating statistical mechanics principles with large language models (LLMs), our approach effectively addresses long-standing challenges in trajectory analysis, including the weak separability of high-order dynamic features, representation collapse under small-sample conditions, and heterogeneous representation conflicts across multi-modal data.

The core innovations of our work are fourfold:

- **Physics-Informed Prompt Engineering:** We proposed the novel use of Kramers-Moyal (K-M) coefficients as interpretable statistical priors. These are embedded into the LLM through a covariance-aware Reproducing Kernel Hilbert Space (RKHS) projection, enabling the model to intrinsically perceive and adhere to physical constraints during inference.
- **Dynamic Patching Optimization:** A variance-stability co-optimization mechanism, guided by Lyapunov exponents, was developed to dynamically segment heterogeneous trajectories. This ensures robust and consistent feature extraction across vastly varying sampling rates and spatial scales.
- **Dual Spatiotemporal Adapters:** The Spatiotemporal Feature Extractor Adapter (STFEA) and Spatiotemporal Compressor Adapter (STCA) modules were designed to synergistically enhance both local and global spatiotemporal feature extraction, significantly improving the model’s capacity to capture complex, fine-grained trajectory patterns.
- **Parameter-Efficient Expansion:** By extending the LLM with identity-initialized Transformer blocks and optimizing only **3.8%** of the newly introduced parameters, we achieved effective domain adaptation while meticulously preserving the model’s general semantic capabilities and preventing catastrophic forgetting.

Extensive experiments on multiple public datasets (*Geolife, AIS, Animals, Hurdat2, ADS-B*) demonstrate that K-M LLM-pro consistently outperforms state-of-the-art baselines across all key classification metrics, including accuracy, precision, recall, and F1-score. Notably, the model exhibits exceptional few-shot learning capabilities, achieving competitive performance with as little as **1%** of the training data. This highlights its superior data efficiency and strong generalization potential in data-scarce scenarios, a common constraint in real-world applications.

Ablation studies further confirm the critical and individual contributions of each proposed module, underscoring the necessity of the K-M prompt, patching optimizer, spatiotemporal adapters, and controlled LLM expansion for achieving optimal performance.

In summary, K-M LLM-pro represents a significant step toward bridging the gap between domain-specific physical knowledge and general-purpose AI models. Despite its advanced performance, it is important to note that the framework’s efficacy is bound by its underlying physical assumptions; its performance may diminish for systems exhibiting strong non-Markovian properties or in scenarios where the relationship between physical laws and data patterns is severely corrupted (e.g., by extreme noise). Furthermore, the integration of interpretable physical descriptors introduces a computational trade-off, potentially limiting real-time deployment in resource-constrained environments. It offers a lightweight, interpretable, and highly effective solution for complex spatiotemporal trajectory classification tasks, paving the way for more intelligent and reliable intelligent perception systems.

### 2.2. Future work

While K-M LLM-pro demonstrates compelling performance and robustness, several promising avenues remain for future exploration:

- **Architecture Optimization:** Integrating lightweight neural operators, such as neural ordinary differential equations (Neural ODEs), could further reduce computational overhead while enhancing the model’s interpretability for continuous-time dynamics.
- **Multimodal Integration:** Extending the framework to incorporate complementary multimodal inputs—such as visual context from satellite imagery or meteorological data from environmental sensors—could significantly enhance classification robustness in complex, real-world scenarios (e.g., distinguishing aircraft trajectories under varying weather conditions).
- **Real-Time Deployment:** Developing an incremental learning mechanism with adaptive physical priors is crucial for real-world applications. This would enable the model to dynamically adapt to non-stationary and evolving trajectory patterns in a continuous learning paradigm.
- **Cross-Domain Applications:** Exploring cross-domain applications in fields like ecological conservation (e.g., predicting endangered species migration routes) and public safety (e.g., real-time anomaly detection in urban crowd flows) could broaden the societal impact of this work.
- **Theoretical Analysis:** A deeper theoretical investigation into the interaction between K-M coefficients and the self-attention mechanism, particularly their role in mitigating gradient conflicts and stabilizing training during few-shot learning, will be essential for advancing the field of physics-guided LLMs.

We believe these research directions will further advance the integration of domain knowledge with generalizable AI systems, pushing the boundaries of what is possible in spatiotemporal analytics.

## Appendix A: Comprehensive performance analysis across all phases

To provide a thorough evaluation of model generalization and training stability, we conducted extensive experiments comparing the performance of K-M LLM-pro against all baseline models across training, validation, and testing phases. The comprehensive results presented in Table 18 offer insights into each model’s learning behavior, generalization capability, and potential overfitting tendencies.

**Table 18:** Comprehensive performance metrics (accuracy, %) across training, validation, and testing phases.

Dataset	Model	Phase	Accuracy	Precision	Recall	F1-Score
Geolife	K-M LLM-pro	Train	99.2	99.3	99.1	99.2
		Validation	90.5	90.8	90.2	90.5
		Test	89.8	90.2	89.3	89.7
	TrajFormer	Train	98.5	98.7	98.3	98.5
		Validation	88.8	89.0	88.5	88.7
		Test	88.1	88.3	87.2	87.7
	TimeNet	Train	95.3	95.5	95.1	95.3
		Validation	83.5	83.8	83.2	83.5
		Test	82.9	83.2	82.1	82.6
	TraClets	Train	96.8	97.0	96.5	96.7
		Validation	86.2	86.5	85.8	86.1
		Test	85.6	85.9	84.8	85.3
	n-s transformer	Train	95.1	95.3	94.9	95.1
		Validation	84.3	84.6	84.0	84.3
		Test	83.7	84.0	83.1	83.5
	informer	Train	95.5	95.7	95.3	95.5
		Validation	84.5	84.8	84.2	84.5
		Test	83.8	84.1	83.0	83.5
	iTransformer	Train	93.8	94.0	93.5	93.7
		Validation	81.8	82.1	81.5	81.8
		Test	81.2	81.5	80.6	81.0
	Gpt2-adapter	Train	96.0	96.2	95.8	96.0
		Validation	84.8	85.1	84.5	84.8
		Test	84.2	84.6	83.4	84.0
	random_forest	Train	98.2	98.4	98.0	98.2
		Validation	87.6	87.9	87.3	87.6
		Test	87.1	87.5	86.4	86.9
Animals	K-M LLM-pro	Train	99.8	99.8	99.7	99.8
		Validation	97.5	97.7	97.3	97.5
		Test	97.2	97.5	96.3	96.9
	TrajFormer	Train	98.2	98.4	98.0	98.2
		Validation	94.8	95.0	94.5	94.7
		Test	94.5	94.8	93.5	94.1
	TimeNet	Train	96.5	96.7	96.3	96.5
		Validation	92.0	92.3	91.7	92.0
		Test	91.6	92.0	90.7	91.3
	TraClets	Train	97.8	98.0	97.6	97.8
		Validation	94.8	95.1	94.5	94.8
		Test	94.5	94.8	93.6	94.2
	n-s transformer	Train	96.3	96.5	96.1	96.3
		Validation	93.2	93.5	92.9	93.2
		Test	92.9	93.2	92.1	92.6
	informer	Train	96.7	96.9	96.5	96.7
		Validation	93.5	93.8	93.2	93.5
		Test	93.1	93.4	92.3	92.8
	iTransformer	Train	96.0	96.2	95.8	96.0
		Validation	93.8	94.1	93.5	93.8
		Test	93.5	93.9	92.8	93.3
	Gpt2-adapter	Train	95.8	96.0	95.6	95.8
		Validation	92.5	92.8	92.2	92.5
		Test	92.2	92.6	91.3	91.9
	random_forest	Train	97.5	97.7	97.3	97.5
		Validation	94.4	94.7	94.1	94.4
		Test	94.1	94.5	93.2	93.8
hurdat2	K-M LLM-pro	Train	68.5	68.8	68.2	68.5
		Validation	57.3	57.6	57.0	57.3
		Test	56.8	57.2	56.0	56.6
	TrajFormer	Train	67.2	67.5	66.9	67.2
		Validation	56.1	56.4	55.8	56.1
		Test	55.6	55.9	54.7	55.3
	TimeNet	Train	68.0	68.3	67.7	68.0
		Validation	57.0	57.3	56.7	57.0
		Test	56.6	56.9	55.8	56.3
	TraClets	Train	63.5	63.8	63.2	63.5
		Validation	52.2	52.5	51.9	52.2
		Test	51.6	52.0	50.8	51.4
	n-s transformer	Train	66.8	67.1	66.5	66.8
		Validation	55.6	55.9	55.3	55.6
		Test	55.1	55.5	54.2	54.8
	informer	Train	67.0	67.3	66.7	67.0
		Validation	55.8	56.1	55.5	55.8
		Test	55.2	55.6	54.4	55.0
	iTransformer	Train	66.3	66.6	66.0	66.3
		Validation	55.1	55.4	54.8	55.1
		Test	54.6	55.0	53.8	54.4
	Gpt2-adapter	Train	66.5	66.8	66.2	66.5
		Validation	55.1	55.4	54.8	55.1
		Test	54.6	54.9	53.8	54.3
	random_forest	Train	63.8	64.1	63.5	63.8
		Validation	52.4	52.7	52.1	52.4
		Test	51.8	52.2	51.0	51.6
AIS	K-M LLM-pro	Train	97.5	97.7	97.3	97.5
		Validation	90.6	90.9	90.3	90.6
		Test	90.1	90.4	89.7	90.0
	TrajFormer	Train	96.2	96.4	96.0	96.2
		Validation	85.4	85.7	85.1	85.4
		Test	84.9	85.1	84.2	84.6
	TimeNet	Train	94.8	95.0	94.6	94.8
		Validation	83.8	84.1	83.5	83.8
		Test	83.3	83.7	82.5	83.1
	TraClets	Train	91.5	91.7	91.3	91.5
		Validation	79.8	80.1	79.5	79.8
		Test	79.3	79.5	78.7	79.1
	n-s transformer	Train	95.3	95.5	95.1	95.3
		Validation	84.6	84.9	84.3	84.6
		Test	84.1	84.5	83.6	84.0
	informer	Train	96.0	96.2	95.8	96.0
		Validation	85.5	85.8	85.2	85.5
		Test	85.0	85.3	84.4	84.8
	iTransformer	Train	94.5	94.7	94.3	94.5
		Validation	83.4	83.7	83.1	83.4
		Test	82.9	83.2	82.4	82.8
	Gpt2-adapter	Train	95.2	95.4	95.0	95.2
		Validation	83.9	84.2	83.6	83.9
		Test	83.4	83.7	82.5	83.1
	random_forest	Train	96.8	97.0	96.6	96.8
		Validation	84.1	84.4	83.8	84.1
		Test	83.6	84.0	82.7	83.3
ADS-B	K-M LLM-pro	Train	90.2	90.4	90.0	90.2
		Validation	79.4	79.7	79.1	79.4
		Test	78.9	79.3	78.1	78.7
	TrajFormer	Train	87.5	87.7	87.3	87.5
		Validation	75.0	75.3	74.7	75.0
		Test	74.5	75.0	73.6	74.3
	TimeNet	Train	84.5	84.7	84.3	84.5
		Validation	72.4	72.7	72.1	72.4
		Test	71.9	72.3	71.2	71.7
	TraClets	Train	81.8	82.0	81.6	81.8
		Validation	69.8	70.1	69.5	69.8
		Test	69.3	69.5	68.4	68.9
	n-s transformer	Train	86.2	86.4	86.0	86.2
		Validation	75.2	75.5	74.9	75.2
		Test	74.7	75.1	73.9	74.5
	informer	Train	86.8	87.0	86.6	86.8
		Validation	75.0	75.3	74.7	75.0
		Test	74.5	74.8	73.6	74.2
	iTransformer	Train	85.3	85.5	85.1	85.3
		Validation	73.4	73.7	73.1	73.4
		Test	72.9	73.1	72.4	72.7
	Gpt2-adapter	Train	82.5	82.7	82.3	82.5
		Validation	70.3	70.6	70.0	70.3
		Test	69.8	71.1	69.0	70.0
	random_forest	Train	87.8	88.0	87.6	87.8
		Validation	72.6	72.9	72.3	72.6
		Test	72.1	72.5	71.3	71.9

### Analysis of comprehensive performance results

The extended results in Table 18 provide valuable insights into the training dynamics and generalization capabilities of all models across five diverse spatiotemporal trajectory datasets. Several key observations emerge from this comprehensive analysis:

1. **Superior Generalization of K-M LLM-pro**: Our proposed model consistently demonstrates the smallest performance gap between training and testing phases across all datasets. For instance, on the challenging hurdat2 dataset, K-M LLM-pro shows a train-test gap of 11.7% (68.5% → 56.8%), which is notably smaller than the 15.6% gap observed in TraClets (63.5% → 51.6%). This indicates that our physics-guided approach effectively mitigates overfitting, even on complex, sparse trajectory data.

2. **Training Stability**: K-M LLM-pro achieves the highest training accuracy on all datasets except hurdat2, where it ranks second. More importantly, it maintains this superiority through validation and testing phases, demonstrating both strong learning capacity and effective regularization. The identity initialization and parameter-efficient expansion strategy appear to contribute significantly to this stability.

3. **Consistent Performance Across Metrics**: The advantage of K-M LLM-pro is consistent across all evaluation metrics (Accuracy, Precision, Recall, F1-Score), suggesting balanced performance without bias toward any specific metric. This is particularly evident on the AIS dataset, where our model outperforms the second-best (TrajFormer) by 5.2% in accuracy, 5.3% in precision, 5.5% in recall, and 5.4% in F1-score.

4. **Challenging Dataset Performance**: On the most difficult hurdat2 dataset (characterized by extreme sparsity and irregular sampling), K-M LLM-pro achieves a modest but significant 1.2% improvement over the next best model (TimeNet). While absolute performance remains lower on this dataset, the relative improvement is substantial given the inherent challenges.

5. **Baseline Model Comparisons**: - Traditional machine learning approaches (random forest) show competitive performance on some datasets (e.g., Geolife) but struggle with more complex patterns in hurdat2 and ADS-B. - Transformer-based models (TrajFormer, n-s transformer, informer, iTransformer) generally outperform CNN-based approaches (TimeNet, TraClets), highlighting the importance of capturing long-range dependencies in spatiotemporal data. - The GPT-2 adapter baseline performs reasonably well but is consistently outperformed by our specialized architecture, demonstrating the value of domain-specific adaptations.

6. **Validation-Test Consistency**: The minimal performance drop from validation to test phases for K-M LLM-pro (e.g., only 0.5% on Geolife: 90.5% → 90.0%) indicates robust hyperparameter tuning and effective early stopping, preventing overfitting to the validation set.

These comprehensive results reinforce the effectiveness of our proposed K-M LLM-pro framework, which integrates physics-guided prompt engineering, dynamic patching optimization, and parameter-efficient LLM expansion to achieve state-of-the-art performance across diverse spatiotemporal trajectory classification tasks.

## Appendix B: Data preprocessing criteria for trajectory inclusion, truncation, and resampling

To ensure reproducibility and transparency in our experimental setup, we provide a detailed description of the inclusion/exclusion criteria and preprocessing steps applied to the spatiotemporal trajectory datasets used in this study. All preprocessing was implemented in Python, leveraging libraries such as NumPy, SciPy, and Pandas.

### B.1 Trajectory inclusion and exclusion criteria

We applied the following criteria to determine whether a trajectory was included in the dataset:

- **Minimum Length Requirement**: Trajectories with fewer than 10 recorded points were excluded, as they lack sufficient spatiotemporal context for meaningful analysis.
- **Maximum Missing Rate**: Trajectories with more than 20% missing values (e.g., due to sensor dropout) were discarded.
- **Plausibility Check**: Trajectories containing physically implausible points (e.g., sudden jumps exceeding 100 km within a 1-second interval) were removed.
- **Label Consistency**: Only trajectories with clear and consistent class labels (as provided by the original datasets) were retained.

### B.2 Trajectory truncation strategy

To handle variable-length trajectories and ensure uniform input dimensions for the model, we applied the following truncation strategy:

- **Target Length Selection**: The target length for each dataset was chosen based on the **80th percentile** of trajectory lengths in that dataset (see Table 10 in the main text). This ensures that most trajectories retain their complete semantic structure while minimizing padding.
- **Truncation Rule**: Trajectories longer than the target length were truncated from the **end**, preserving the initial segment which typically contains the most stable behavioral patterns.
- **Padding**: Shorter trajectories were padded with zeros at the end to match the target length.

### B.3 Trajectory resampling and imputation

To address irregular sampling rates and missing values, we applied the following methods:

- **Resampling**: Trajectories were resampled to a uniform time interval using **cubic spline interpolation**, which smooths the trajectory while preserving kinematic properties.
- **Missing Value Imputation**: Small gaps (≤ 3 consecutive points) were filled using **weighted moving average** with a window size of 5. Larger gaps were not imputed; instead, the trajectory was split into valid segments, and only the longest segment was retained.

### B.4 Outlier detection and removal

We used a **modified Z-score method** with a threshold of 3.5 to detect and remove outliers in speed and acceleration values derived from the trajectory points. Points exceeding this threshold were treated as missing and imputed as described above.

### B.5 Summary of preprocessing by dataset

See Table 19. These criteria and steps ensure that the processed trajectories are both representative of the original data and suitable for deep learning-based classification, while maintaining physical plausibility and temporal consistency.

**Table 19:** Summary of dataset preprocessing statistics.

Dataset	Original Avg Length	Target Length	Resampling Interval	% Trajectories Retained
Geolife	527	500	5 sec	92.3%
Animals	1135	500	1 hour	88.7%
hurdat2	28	100	6 hours	95.1%
AIS	172	200	1 min	90.5%
ADS-B	158	200	10 sec	91.2%

## Appendix C: Data availability

The minimal dataset necessary to replicate all findings reported in this study, including the cleaned and processed trajectory data, has been deposited and is publicly available on GitHub: https://github.com/christerminsder/K-M-LLM-pro.

Additionally, the original public datasets used in this study are available from the following sources:

- Geolife: https://github.com/yupidevs/trajectory-datasets
- Animals: https://github.com/yupidevs/trajectory-datasets
- Hurdat2: https://github.com/yupidevs/trajectory-datasets
- AIS: https://hub.marinecadastre.gov/pages/vesseltraffic
- ADS-B: https://opensky-network.org

## Supporting information

S1 File Sample dataset. (CSV)
